# Risk Perception About HIV Among University Students in One of the Last Hotspots for HIV Transmission in Europe

**DOI:** 10.1007/s44197-023-00151-y

**Published:** 2023-09-20

**Authors:** Marija Milic, Tatjana Gazibara, Jelena Dotlic, Natasa Katanic, Jelena Filimonovic, Katarina Mitic, Marijan Bakic, Igor Galic, Slavica Aksam, Dusica Kocijancic Belovic, Melchizedek Nyakundi Mokaya, Jasmina Stevanovic

**Affiliations:** 1 Department of Epidemiology, Faculty of Medicine, University of Pristina Temporarily Settled in Kosovska Mitrovica, Anri Dinana bb, 38228 Kosovska Mitrovica, Serbia; 2 Institute of Epidemiology, Faculty of Medicine, University of Belgrade, Belgrade, Serbia; 3 Clinic for Obstetrics and Gynecology, Clinical Center of Serbia, University of Belgrade, Belgrade, Serbia; 4 Medical Faculty, University of Belgrade, Belgrade, Serbia; 5 Department of Infectious Diseases, Faculty of Medicine, University of Pristina Temporarily Settled in Kosovska Mitrovica, Kosovska Mitrovica, Serbia; 6 Emergency Relief Project “Solidarity”, SOS Children’s Villages Serbia, Kraljevo, Serbia; 7 Department of Epidemiology, Institute of Public Health of Montenegro, Podgorica, Montenegro; 8 Ministry of Interior and Coordination of National Government, State Department of Correctional Services, Probation and Aftercare Services, Nairobi, Kenya

**Keywords:** HIV, Self-perceived risk, Knowledge of HIV prevention and transmission, Students, Kosovo

## Abstract

**Background:**

HIV testing in the Northern Kosovo province is challenging, because the infrastructure is being rebuilt after the ethnic conflict. The purpose of this research was to examine self-perceived risk for acquiring HIV infection and factors associated with risk assessment among university students.

**Methods:**

Students completed a questionnaire on socio-demographic data, knowledge about HIV prevention and transmission, attitudes toward people living with (PLHIV) and self-perceived risk for HIV infection. The self-perceived risk was categorized as low, unknown and high.

**Results:**

The majority of students (72.5%) assessed their risk as low, 8.5% assessed their risk as high and 19.1% did not know their risk. Compared to low self-perceived risk, high self-perceived HIV risk was associated with being male, having lower knowledge about HIV prevention, less strong Segregation and protection attitude toward PLHIV, stronger Ignorance and indifference attitude toward PLHIV and positive opinion about gays/lesbians. Students who perceived own risk for acquiring HIV as high had lower knowledge about HIV transmission and prevention. However, those who were previously tested for HIV, despite their poorer knowledge about HIV prevention, assess their HIV-related risk as low.

**Conclusions:**

Students assessed their risk of HIV infection mostly as low. Still, lower knowledge of HIV prevention has been consistently associated with a high and unknown risk of HIV. Moreover, being ignorant and indifferent about PLHIV was associated with increased self-perceived HIV risk. These findings highlight the need for continuous specialized HIV-related education to reduce fear and stigma of PLHIV and HIV testing as well as risky behaviors.

**Supplementary Information:**

The online version contains supplementary material available at 10.1007/s44197-023-00151-y.

## Introduction

Practice of risky behaviors, particularly sexual intercourse without a condom, has been related to the increase in global prevalence of HIV/AIDS among university students in recent years [1]. In the late 1990s, the importance of HIV risk perception has been addressed as the key component to reduce one’s exposure to health risks [2, 3]. This is because adequate estimation of risk is a compelling factor to change risky behaviors, which may reduce the incidence of HIV [2, 3]. Thus, self-perceived risk for HIV has the potential to influence the course of the HIV epidemic [4, 5].

Previous studies suggested that the perception of HIV risk in the university student population is generally low [6, 7]. In fact, it has been observed that students from countries, where the HIV prevalence is low, have a sense of false security and believe that they do not come in contact with people living with HIV (PLHIV) [8]. It seems that a lack of information and awareness about HIV infection make most young adults negligent about the true problem of HIV transmission and their individual risk [9, 10].

The prevalence of HIV in Serbia is low (0.1% with a 100 newly diagnosed cases each year per seven million inhabitants), but it remains one of the highest in the Southeastern Europe [11]. People aged 20–29 years account for one-third of newly diagnosed PLHIV (incidence rate 6.38/100,000) [12]. In addition, the prevalence is quite high in special population groups (up to 8% for men who have sex with men, sex workers and injecting drug users) [13]. The low rates of testing suggest that perception of HIV risk among young adults in Serbia is expectedly low. The HIV testing in the Northern Kosovo province is even more challenging.

The territory of Kosovo has been disputed for the past two decades between the people of Serbian and Albanian nationality. Following the civil war in the 1990s and the declaration of Kosovo independence in the 2000s, the province of Kosovo has been divided into the Northern Kosovo (inhabited by the Serbs) and the Southern Kosovo (inhabited by the Albanians). Moreover, in the Southern Kosovo there are Serbian settlements (enclaves), where approximately 70,000 people still reside. The Northern and Southern parts of Kosovo remain entirely separate entities regarding their political, economic, social and cultural backgrounds. Although the civil war ended, the ethnic tensions continue to disrupt everyday living, so foreign military troops have been stationed throughout Kosovo since 1999 as peacekeepers [13].

Because of the ongoing clashes that frequently escalate, the infrastructure and the sense of normalcy in the Northern Kosovo cannot be fully rebuilt despite the efforts to improve living conditions. Some of the factors that are deemed as the contributors to the potential HIV spread in this region include the presence of military, high turnover of different non-governmental organizations (NGOs), high unemployment rate, migrations, younger resident population, sex work and illegal drug trade [13].

The largest town in the Northern Kosovo (Kosovska Mitrovica) in its northern part is inhabited by approximately 30,000 people of Serbian nationality. Although the University is situated in Kosovska Mitrovica, the town is not as developed as other cities of the same size in Serbia. Access to health care in the Northern Kosovo is universal. There is only one HIV-related counseling center which belongs to the Students’ health center (primary and secondary health care delivery institution) and is accessible to all free of charge [14]. Because of a small and interconnected population, the center is visited by few people, as the very visit is associated with being branded as “infected”. As HIV is still being heavily stigmatized [11, 15] in Kosovo most people refrain from visiting even this center. The HIV stigma in the region is based on the notion that only injecting drug users, sex workers, and men who have sex with men (particularly those who have encounters with the members of the foreign military and NGOs) are the drivers of the HIV transmission. For this reason, few people actually take the test, and therefore, currently, there are no precise data regarding incidence of HIV in northern Kosovo province [16]. Bearing in mind all mentioned above, the Kosovo province has been labeled as a setting at increased risk of HIV infection [13].

The purpose of this study was to examine self-perceived risk for HIV infection and factors associated with risk assessment among Serbian university students in the Northern Kosovo province.

## Methods

### Setting and Participants

We invited students from the University of Pristina temporarily seated in Kosovska Mitrovica, northern Kosovo province. At the time of survey, approximately 5,000 students attended the University. Students were recruited from the first and fourth study years to take into consideration both younger and older students who are at the beginning and the end of their schooling. Students from all ten schools from the University were included (biomedical: medicine, dentistry and nursing; art; economy; law; natural sciences: biology, geography, mathematics, physics and chemistry; technical sciences: technology, construction and engineering; social sciences: philosophy, sociology, psychology and languages; agriculture; sports and pedagogy). All the classes are held in the Serbian language given that the Serbian ethnic group comprises the majority in the northern part of Kosovska Mitrovica.

The process of data collection was carried out throughout the summer semester in 2014 on two random days (Mondays and Thursdays). To approach various students, convenience sampling was applied in all the classrooms, where mandatory classes were taught, which minimized an error due to chance. We gathered data over the course of 1 week in each of the schools. A total of 1225 students were approached to participate. Of these, 1017 agreed to participate (response rate 82.02%). This sample of students represents 28.9% of all 3524 first- and fourth-year students from the University.

Students provided written informed consent for participation before taking the survey. We received the approval to conduct the study from the Ethics Committee of the School of Medicine, University of Pristina temporally seated in Kosovska Mitrovica (Approval No. 09-1608-1, issued on October 29, 2013).

### Instrument

Data for this study were collected using an anonymous questionnaire. This questionnaire was first developed and validated for use in the general population as one of the outcomes of the large national project supported by the Global Fund for the Fight against AIDS, Tuberculosis and Malaria (GFATM) (UNAIDS, 2014). The questionnaire was adapted for the Serbian population during the project “Strengthening HIV Prevention and Care for the Groups Most Vulnerable to HIV/AIDS” (Grant no. SER-809-G04-H and SER-809-G05-H; project funder: The Global Fund for the Fight against AIDS, Tuberculosis and Malaria; UNAIDS, 2014; The Global Fund, 2019; Ministry of Health of Serbia, 2013). To adjust the items for the student population, we included the questions about place of residence during schooling, visiting nightclubs, alcohol intake and use of illicit drugs.

### Observed Outcome

Students’ perception of their own risk for acquiring HIV was examined by a question “How do you rate your own risk to contract HIV?”. The answers were graded as follows: (1) very low, (2) low, (3) I do not know, (4) high, (5) very high. The two former answers (low and very low perceived risk) were combined into a category “low self-perceived risk”. The answer “I do not know” was categorized as “unknown risk”. The two latter answers (high and very high perceived risk) were combined into a category of “high self-perceived risk”.

### Covariates

Age, gender, year at school, type of school program (biomedical; others), type of residence during schooling (student dormitory; alone in a rented or privately owned property; with parents), relationship status (single; in a relationship) and doing paid work while studying comprised demographic characteristics.

Specific sources of information about HIV that students used to receive information about HIV included media, internet, medical sources, university professors, friends and special (peer) education about HIV.

The knowledge about HIV was examined using the Knowledge scale with 14 items regarding the modes of HIV transmission (hand shaking, sexual intercourse, via cutlery, hygiene products, swimming in pools, sea or rivers, equipment for drug injection, mother to child, etc.), prevention and current therapy (Supplemental Table S1). Students were able to choose three possible answers: “no”, “I do not know” and “yes”. The answers were scored as follows: one point for an incorrect answer, two points for being unsure/not knowing the answer and three points for a correct answer. The sum of all points was observed as the Knowledge score. Higher scores suggested a better knowledge about HIV. Using the exploratory factor analysis (EFA), we found that the Knowledge scale was composed of two domains: one about transmission (Knowledge of transmission) and other about prevention (Knowledge of prevention). These two domains explained 51% of variance (Supplemental Table S1). These two knowledge domains were analyzed separately. Possible range for Knowledge of transmission domain was 8–24 and for Knowledge of prevention 6–18.

The attitudes toward people living with (PLHIV) were examined using the Attitude scale with 17 statements. These statements included questions regarding different PLHIV rights and life conditions (Supplemental Table S2). Possible answers were: “I do not agree”, “I am not sure” and “I agree”. The answers were scored as follows: one point for negative answer, two points for not being sure and three points for positive answer. The sum of all points was observed as the Attitude score. Higher scores suggested stronger support and positive attitude about PLHIV. Based on the results of EFA, we observed that the Attitude scale consisted of three domains explaining 43% of variance (Supplemental Table S2). The three domains were entitled “Support and treatment”, “Segregation and protection” and “Ignorance and indifference” attitudes. Support and treatment domain encompassed attitudes of equal rights for PLHIV. Segregation and protection domain included the feelings of fear of PLHIV and intentions to isolate PLHIV from the HIV-negative population. Items from the Ignorance and indifference domain were related to the lack of both knowledge and interest in issues regarding HIV and PLHIV. These three attitude domains were analyzed separately. Possible range for Support and treatment domain was 5–15, for Segregation and protection domain 7–21 and for Ignorance and indifference 5–15.

We also asked whether students had previously taken the HIV test and whether they were interested in taking the test. Awareness and interest in HIV testing were assessed by asking the students about their position in case they wanted to take the test (possible answers: I know where to take the test/I do not know where to take the test, but I know whom to ask/the test cannot be taken in our country/I do not know, I do not care). Circling one of former two answers was classified as “positive approach to HIV testing”. Circling one of latter two answers was classified as “negative approach to HIV testing”.

Students’ opinion about high-risk groups for contracting HIV was related to persons who inject drugs, gays/lesbians and sex workers (answers were: it is their right; I avoid them; I feel that I need protection from them; I do not think about them).

Furthermore, we asked the students whether they previously had contact with PLHIV and whether they would change the level of contact after learning about one’s HIV status (possible answers: same level of contact, decrease the contact, stop the contact, not sure what to do).

Finally, health risk behaviors related to HIV included items about frequenting nightclubs (a proxy measure for different risky behaviors that occur more frequently in nightclubs), level of alcohol intake (heavy/binge drinking—5 or more drinks on one occasion; moderate drinking—2 or less drinks on one occasion; not drinking alcohol), use of illicit drug/opioids use (yes/no), number of sexual partners in the past 12 months (one or less, more than one), condom use at last sex, condom use with casual (non-committed) partner (always, sometimes, i.e., when you have it and feel like it or only as contraception during fertile days, with new partner only and not in a stable relationship, never, no sexual intercourses) and having and treating sexually transmitted infections (STIs) in the past 12 months.

### Statistical Analysis

Data were analyzed using the SPSS 21 for Windows. The Kolmogorov–Smirnov test suggested the normality of data distribution. To examine the association of the investigated parameters with self-perceived risk for contracting HIV, we performed the multinomial regression analysis as our outcome variable had three possible responses (low, unknown and high self-perceived risk). The discriminant analysis was used to determine the parameters that separate two or more groups, i.e., outcome categories by constructing discriminatory functions. To assess potential interactions between factors associated with self-perceived risk for contracting HIV infection, data were tested for the moderation analysis. A detailed description of each analysis is provided in the Supplemental material.

## Results

### Sample Description

A total of 1017 students participated in the study (44.6% were males and 55.4% females). More than one-half of students (53.8%) were in a relationship, 30.3% of them studied health-related disciplines and 62.3% were in the first faculty year. The mean ± standard deviation (SD) age of examined students was 21.3 ± 3.5 years. The majority of students informed themselves about HIV through internet (81%), never used drugs/opioids, went clubbing on weekends and reported moderate alcohol intake (Tables 1, 2, 3).

**Table 1:** Descriptive data of students’ according to self-perceived HIV risk

Parameters		Perception of risk for acquiring HIV		
		High	Unknown	Low
Students’ age	Mean	21.11	21.09	21.32
	Standard deviation	3.23	3.36	3.56
Knowledge about HIV transmission score	Mean	18.26	18.04	18.81
	Standard deviation	2.31	2.62	2.14
Knowledge about HIV prevention score	Mean	13.41	13.65	14.27
	Standard deviation	1.82	1.94	1.87
Support and treatment attitude toward PLHIV score	Mean	12.58	13.51	13.49
	Standard deviation	3.05	2.75	2.511
Ignorance and indifferent attitude toward PLHIV score	Mean	9.73	10.14	10.07
	Standard deviation	2.01	1.86	1.64
Segregation and protection attitude toward PLHIV score	Mean	8.27	8.47	7.84
	Standard deviation	2.22	2.06	1.78

*PLHIV* persons living with HIV

**Table 2:** Students’ sociodemographic, medical and HIV information-related data

Parameters	Perception of risk for acquiring HIV					
	High		Unknown		Low	
	Number	Percent	Number	Percent	Number	Percent
Students’ gender						
Male	50	58.1	90	46.4	314	42.6
Female	36	41.9	104	53.6	423	57.4
School group						
Biomedical	30	34.9	55	28.4	223	30.3
Others	56	65.1	139	71.6	514	69.7
Study year						
First	56	65.1	130	67.0	448	60.8
Fourth	30	34.9	64	33.0	289	39.2
Extra activities						
Works and studies	7	8.1	13	6.7	39	5.3
Just student	79	91.9	181	93.3	698	94.7
Relationship status						
Single	33	38.4	96	49.5	341	46.3
Not single	53	61.6	98	50.5	396	53.7
Residence during schooling						
Student dormitory	28	32.6	55	28.4	200	27.1
Alone	42	48.8	83	42.8	316	42.8
With parents	16	18.6	56	28.9	221	30.0
STI in last year						
Yes/had and treated	1	1.2	8	4.1	23	3.1
No	85	98.8	186	95.9	714	96.9
Contact with PLHIV						
Yes/had	6	7.0	3	1.5	28	3.8
No	80	93.0	191	98.5	709	96.2
Previous HIV testing						
Yes/had	7	8.1	9	4.6	39	5.3
No	79	91.9	185	95.4	698	94.7
Media for information						
Yes	63	73.3	111	57.2	504	68.4
No	23	26.7	83	42.8	233	31.6
Internet for information						
Yes	69	80.2	146	75.3	613	83.2
No	17	19.8	48	24.7	124	16.8
Medical information						
Yes	53	61.6	134	69.1	457	62.0
No	33	38.4	60	30.9	280	38.0
Professors information						
Yes	47	54.7	86	44.3	379	51.4
No	39	45.3	108	55.7	358	48.6
Information from friends						
Yes	60	69.8	118	60.8	460	62.4
No	26	30.2	76	39.2	277	37.6
Special HIV education						
Yes	23	26.7	56	28.9	218	29.6
No	63	73.3	138	71.1	519	70.4

*STI* sexually transmitted infections, *PLHIV* persons living with HIV

**Table 3:** Students’ opinions and risky behaviors according to self-perceived HIV risk

Parameters	Perception of risk for acquiring HIV					
	High		Unknown		Low	
	Number	Percent	Number	Percent	Number	Percent
Approach to HIV testing						
Positive	60	69.8	114	58.8	547	74.2
Negative	26	30.2	80	41.2	190	25.8
Actions after contacting PLHIV						
Not sure what to do	16	18.6	41	21.1	151	20.5
Stop contact	14	16.3	32	16.5	52	7.1
Less contact	23	26.7	61	31.4	193	26.2
Same contact	33	38.4	60	30.9	341	46.3
Opinion on drug addicts						
It is their right	13	15.1	42	21.6	177	24.0
I avoid them	32	37.2	58	29.9	229	31.1
I need protection from them	25	29.1	63	32.5	244	33.1
Do not think of them	16	18.6	31	16.0	87	11.8
Opinion on gays/lesbians						
It is their right	24	27.9	40	20.6	255	34.6
I avoid them	17	19.8	67	34.5	231	31.3
I need protection from them	24	27.9	43	22.2	126	17.1
Do not think of them	21	24.4	44	22.7	125	17.0
Opinion on sex workers						
It is their right	24	27.9	57	29.4	254	34.5
I avoid them	25	29.1	49	25.3	192	26.1
I need protection from them	15	17.4	38	19.6	112	15.2
Do not think of them	22	25.6	50	25.8	179	24.3
Clubbing frequency						
Every night	9	10.5	10	5.2	16	2.2
Couple times/week	11	12.8	31	16.0	122	16.6
During weekends	46	53.5	110	56.7	444	60.2
Do not go clubbing	20	23.3	43	22.2	155	21.0
Alcohol drinking						
Heavy/binge drinking	7	8.1	6	3.1	52	7.1
Moderate drinking	51	59.3	139	71.6	473	64.2
I do not drink	28	32.6	49	25.3	212	28.8
Opioids use						
Yes/use	2	2.3	7	3.6	38	5.2
No	84	97.7	187	96.4	699	94.8
Sex partners in last year						
More than one	30	34.9	53	27.3	182	24.7
One or less	56	65.1	141	72.7	555	75.3
Condom use in last sex						
Yes	49	57.0	90	46.4	337	45.7
No	37	43.0	104	53.6	400	54.3
Condom use with casual partner						
Always	36	41.9	71	36.6	288	39.1
Sometimes	14	16.3	46	23.7	120	16.3
With new partner	6	7.0	11	5.7	50	6.8
Never	6	7.0	11	5.7	36	4.9
No sex	24	27.9	55	28.4	243	33.0

heavy/binge drinking—5 or more drinks on one occasion; moderate drinking—2 or less drinks on one occasion; clubbing during the week—from Sunday to Thursday; weekend clubbing—Friday and Saturday only; condom sometimes—when you have it and feel like it or only as contraception during fertile days; condom with new partner—only casual partners and not in a stable relationship

*PLHIV* persons living with HIV

The majority of the investigated students (70.9%) had positive attitude toward the HIV testing; however, 5.4% have actually taken the test. Most students (74.9%) believed that they had no need to be tested for HIV. During the past year almost 75% of students had one or less sexual partners in the previous year, while only 4.5% reported having and treating STIs. Around 40% of the study participants confirmed that they used condom every time they had sexual intercourse. However, more than one half of students did not use condom at last sexual intercourse, while almost 32% of students had never used condom with a casual partner (Tables 1, 2, 3).

### Knowledge About HIV

The mean ± SD of students for Knowledge of transmission domain was 18.62 ± 2.28 (range 10.0 to 24.0) and for Knowledge of prevention domain it was 14.08 ± 1.91 (range 8.0 to 18.0). Despite the high mean knowledge scores, there were 292 (28.71%) students who had Knowledge of transmission below average, while 379 (37.27%) students had Knowledge of prevention score under the mean value for the total sample. In our study, there were no students who had zero scores on both Knowledge of transmission and Knowledge of prevention domains.

### Attitude Toward Persons Living with HIV

The mean ± SD of Support and treatment attitude domain was 8.01 ± 1.91 (range 5.0–15.0), of Segregation and protection attitude domain was 13.42 ± 2.61 (range 7.0–21.0) and of Ignorance and indifference attitude domain it was 10.06 ± 1.72 (range 5.0–15.0). In our sample, there were no students with absolutely negative attitude toward PLHIV and 556 (54.67%) students had positive attitudes on PLHIV with scores above the mean in Support and treatment domain. Moreover, 393 (38.64%) students had scores below mean in the Ignorance and indifference domain and 353 (34.71%) had scores below mean in the Segregation and protection domain. Still, only 29 (2.85%) students had scores over 13 in Support and treatment domain.

### Self-Perceived Risk for Contracting HIV

In our sample of students from the Northern Kosovo province 737 (72.5%) students assessed their risk for contracting HIV as low. Risk for HIV was assessed as high by only 86 (8.5%) students, while 194 (19.1%) students were unsure and unable to properly asses their HIV risk (and were labelled as having unknown risk). Correlations of examined students’ socio-demographic characteristics, behaviors, positions and experiences with their self-perceived risk for HIV infection are presented in Supplemental Table S1.

### Multinomial Regression Model

Multinomial regression models for factors that might be associated with students’ self-perceived risk for contracting HIV were significant (*χ*^2^ = 118.747; *p* = 0.001; Pearson goodness-of-fit = 0.216; *R*^2^ Nagelkerke = 0.141; classification = 73.8%).

Compared with students who assessed their risk for acquiring HIV as low, students who assessed their risk for HIV as high were more likely to be male, have lower knowledge about HIV prevention, lower scores on Segregation and protection attitude and higher score on Ignorance and indifference attitude toward PLHIV and have positive opinion about gays/lesbians.

Compared with students who assessed their risk for acquiring HIV as low, students who perceived their risk for HIV as unknown were more likely to have lower knowledge about both HIV transmission and prevention, higher scores on Ignorance and indifference attitude, negative approach to HIV testing, be unsure what to do or reduce the contact after learning about one’s HIV status and have positive opinion about gays/lesbians (Table 4).

**Table 4:** Factors associated with high and unknown self-perceived risk for acquiring HIV: results of multinomial regression (the reference category is low self-perceived HIV risk)

Parameters	*B*	Wald	*p*	OR	CI low	CI high
High risk						
Intercept	1.617	1.120	0.290			
Knowledge transmission	− 0.034	0.407	0.523	0.967	0.872	1.072
Knowledge prevention	− 0.165	6.665	**0.010**	0.848	0.748	0.961
Support-treatment at	− 0.104	2.028	0.154	0.901	0.781	1.040
Segregation-protection At	− 0.125	6.211	**0.013**	0.882	0.799	0.974
Ignorance-indifference At	0.170	5.939	**0.015**	1.186	1.034	1.360
Male gender	0.623	4.796	**0.029**	1.864	1.068	3.256
Been tested for HIV	0.452	0.958	0.328	1.572	0.635	3.890
Positive testing approach	− 0.042	0.024	0.877	0.959	0.568	1.621
Position after learning one’s HIV + status not sure	0.014	0.002	0.968	1.014	0.518	1.982
Position after learning one’s HIV + status negative	0.448	1.177	0.278	1.565	0.697	3.517
More sex partners	0.239	0.691	0.406	1.270	0.723	2.231
Condom last intercourse	0.445	2.760	0.097	1.560	0.923	2.638
Condom always	− 0.362	1.078	0.299	0.696	0.352	1.379
Condom sometimes	− 0.126	0.103	0.749	0.882	0.408	1.904
Condom with new partner	− 0.175	0.110	0.740	0.840	0.299	2.355
Condom never	0.110	0.043	0.836	1.117	0.394	3.167
Positive on gay/lesbians	− 0.299	0.790	0.374	0.741	0.383	1.435
Negative on gay/lesbians	− 1.113	9.247	**0.002**	0.329	0.160	0.673
Unknown risk						
Intercept	0.695	0.393	0.531			
Knowledge transmission	− 0.084	4.884	**0.027**	0.919	0.853	0.991
Knowledge prevention	− 0.095	4.116	**0.042**	0.909	0.830	0.997
Support-treatment at	− 0.010	0.038	0.846	0.990	0.894	1.096
Segregation-protection At	0.013	0.134	0.714	1.013	0.945	1.086
Ignorance-indifference At	0.105	4.290	**0.038**	1.110	1.006	1.226
Male gender	− 0.008	0.002	0.968	0.992	0.661	1.488
Been tested for HIV	0.213	0.280	0.596	1.238	0.562	2.727
Positive testing approach	− 0.460	6.480	**0.011**	0.631	0.443	0.900
Position after learning one’s HIV + status not sure	0.172	0.522	0.470	1.187	0.745	1.891
Position after learning one’s HIV + status negative	0.754	6.265	**0.012**	2.126	1.178	3.839
More sex partners	0.036	0.028	0.867	1.037	0.677	1.588
Condom last intercourse	0.087	0.200	0.655	1.091	0.746	1.595
Condom always	0.078	0.096	0.757	1.081	0.660	1.770
Condom sometimes	0.459	3.246	0.072	1.582	0.961	2.606
Condom with new partner	0.054	0.019	0.889	1.056	0.492	2.267
Condom never	0.179	0.199	0.656	1.196	0.545	2.624
Positive on gay/lesbians	− 0.529	4.239	**0.039**	0.589	0.356	0.975
Negative on gay/lesbians	− 0.113	0.228	0.633	0.893	0.560	1.423

Significant variables are bolded

*At* attitude

### Discriminant Analysis

When the influence of the two knowledge scores and three attitude scores was tested in relation to self-perceived risk for acquiring HIV, we obtained 2 significant discriminatory functions (p1 = 0.001; variance = 85.1%; p2 = 0.041; variance = 14.9%). According to the largest group centroids, the first significant function discriminates low risk (high = − 0.464; unknown = − 0.347; low = 0.146), while second discriminates unknown from other self-perceived risk options (high = − 0.264; unknown = 0.145; low = − 0.007).

This means that students who perceive own risk for acquiring HIV as low score high on both knowledge scores (prevention and transmission) and score low on Segregation and protection attitude domain. Furthermore, students who perceive their own risk for acquiring HIV as unknown have higher scores on Support and treatment attitude and Ignorance and indifference attitude (Table 5).

**Table 5:** Discriminant analysis – functions of associations between self-perceived HIV risk and HIV related knowledge and attitude scores of investigated students

Parameters	Function 1	Function 2
Knowledge on HIV prevention score	0.703^*^	0.106
Knowledge on HIV transmission score	0.572^*^	− 0.409
Support and treatment attitude	− 0.557^*^	0.420
Segregation and protection attitude	0.241	0.801^*^
Ignorance and indifference attitude	0.086	0.561^*^

Variables in the table are ordered by absolute size of correlation within function. Bold and ^*^ represent largest absolute correlation between each variable and any discriminant function

### Moderation Analysis

To examine potential interactions between the variables associated with students self-perceived risk, the moderation analysis was performed for both knowledge about HIV transmission (*R* = 0.279; *R*^2^ = 0.078; MSE = 0.376; *F* = 4.201; *p* = 0.001) and about prevention (*R* = 0.256; *R*^2^ = 0.065; MSE = 0.381; *F* = 3.485; *p* = 0.001).

A significant interaction was observed only for the product term Knowledge of prevention x been tested for HIV. We observed that the lower knowledge about prevention and having been tested, when observed separately, were associated with higher self-perceived risk for HIV. However, the interaction between these two variables suggests that students who have the experience of previous HIV testing despite their poorer knowledge about HIV prevention assess their HIV-related risk as low (these two variables have sort of “antagonistic effect”). Moreover, scoring lower on Segregation and protection and higher on Support and treatment attitudes toward PLHIV as well as positive opinion about gays/lesbians, were also associated with higher self-perceived risk for HIV (Table 6).

**Table 6:** Results of moderation analysis (interaction) of factors associated with higher self-perceived risk for acquiring HIV

Parameters	Coefficient	SE	*t*	*p*	LLCI	ULCI
(Constant)	− 1.5753	1.1581	− 1.3603	0.1740	− 3.8478	0.6972
Been tested before	1.8024	0.5904	3.0530	**0.0023**	0.6439	2.9609
KnPrev	0.2694	0.0783	3.4411	**0.0006**	0.1158	0.4231
Interaction	− 0.1172	0.0402	− 2.9194	**0.0036**	− 0.1960	− 0.0384
IgnInAt	0.0196	0.0123	1.5971	0.1106	− 0.0045	0 .0438
SegPrAt	0.0221	0.0082	2.6968	**0.0071**	0.0060	0.0382
SupTrAt	− 0.0464	0.0115	− 4.0336	**0.0001**	− 0.0690	− 0.0238
Students’ gender	0.0852	0.0482	1.7669	0.0776	− 0.0094	0.1799
Students’ age	0.0014	0.0075	0.1819	0.8557	− 0.0134	0.0162
Relationship status	− 0.0618	0.0401	− 1.5389	0.1242	− 0.1405	0.0170
Sex partners number	0.0371	0.0503	0.7383	0.4605	− 0.0615	0.1357
Condom at last sex	0.0559	0.0427	1.3089	0.1909	− 0.0279	0.1396
Condom use ever	− 0.0066	0.0135	− 0.4873	0.6262	− 0.0330	0.0199
Testing approach	− 0.0746	0.0440	− 1.6945	0.0905	− 0.1610	0.0118
After contact action	0.0110	0.0173	0.6358	0.5251	− 0.0229	0.0449
Opinion gay/lesbian	− 0.0439	0.0221	− 1.9845	**0.0475**	− 0.0873	− 0.0005
Opinion drug users	− 0.0109	0.0235	− 0.4659	0.6414	− 0.0570	0.0351
Opinion sex workers	0.0048	0.0201	0.2404	0.8101	− 0.0345	0.0442
(Constant)	2.8551	1.3552	2.1067	0.0354	0.1957	5.5144
Had HIV test	− 0.4075	0.6896	− 0.5908	0.5548	− 1.7608	0.9458
KnTrans	− 0.0256	0.0714	− 0.3584	0.7201	− 0.1656	0.1145
Interaction	0.0260	0.0365	0.7132	0.4759	− 0.0455	0.0975
IgnInAt	0.0207	0.0124	1.6662	0.0960	− 0.0037	0.0450
SegPrAt	0.0244	0.0083	2.9539	0.0032	0.0082	0.0406
SupTrAt	− 0.0528	0.0114	− 4.6226	0.0000	− 0.0752	− 0.0304
Students’ gender	0.0834	0.0487	1.7142	0.0868	− 0.0121	0.1789
Students’ age	0.0006	0.0076	0.0769	0.9387	− 0.0144	0.0156
Relationship status	− 0.0588	0.0404	− 1.4555	0.1458	− 0.1381	0.0205
Sex partners number	0.0450	0.0507	0.8875	0.3750	− 0.0545	0.1445
Condom at last sex	0.0711	0.0428	1.6606	0.0971	− 0.0129	0.1552
Condom use ever	− 0.0119	0.0135	− 0.8784	0.3800	− 0.0384	0.0147
Testing approach	− 0.0734	0.0444	− 1.6521	0.0988	− 0.1606	0.0138
After contact action	0.0161	0.0174	0.9270	0.3541	− 0.0180	0.0501
Opinion gay/lesbians	− 0.0484	0.0222	− 2.1816	0.0294	− 0.0920	− 0.0049
Opinion drug users	− 0.0116	0.0236	− 0.4929	0.6222	− 0.0580	0.0347
Opinion sex workers	0.0065	0.0202	0.3214	0.7479	− 0.0332	0.0462

Significant variables are bolded

*IgnInAt* Ignorance and indifferent attitude, *SegPrAt* Segregation and protection attitude, *SupTrAt* Support and treatment attitude

## Discussion

This study suggests that most students perceived their risk for acquiring HIV as low. The knowledge score about prevention and transmission of HIV was below mean in one-third of students in our study. Lower knowledge score about HIV prevention was consistently associated with unknown and high self-perceived risk for acquiring HIV. Previous experience with HIV testing combined with low knowledge seem to change the direction of this association, i.e., students who have low knowledge about HIV prevention who took the HIV test assess their risk as low. This means that previous testing experience plays an important role in the assessment of risk. Finally, stronger Ignorant and indifferent attitude toward PLHIV was associated with increased risk for HIV.

Accurate assessment of risk for HIV is important, because it can direct subsequent preventive behavior [17, 18]. Previous studies examining HIV-related risk perception pointed out that students and young people generally perceive their risk of HIV infection as low and, therefore, frequency practice sexual intercourse without condom [6, 7, 19]. Engaging in unprotected sexual intercourse, while knowingly exposing oneself to HIV could be related to the inability to effectively negotiate use of condom [20], feeling invulnerable and "better than average" [8] or being under influence of alcohol or drugs [21]. Engaging in sexual activity under the influence of alcohol is perceived as particularly risky [22]. Despite this, it is also interpreted as a positive experience that favors sexual enhancement and disinhibition [22], especially during casual and unprotected sexual encounters commonly referred to as ‘hook-ups’ [23].

Based on the study results, lower knowledge about HIV prevention appears to have more influence on high self-perceived risk, rather than knowledge about HIV transmission. Some studies found that college students continue to lack knowledge about HIV transmission as well as about sexual health [24, 25]. However, other authors reported that more than two-thirds of students do not use condom at sexual intercourse despite being knowledgeable about HIV prevention [26]. More so, some students do not practice condom use regardless of their perception of moderate-to-high risk of HIV exposure [27]. The findings from this study suggest that further sexual education is needed not only for university students, but that this type of education should begin as early as adolescence.

Nevertheless, this might be an issue in the Kosovo province, because sexual education overall is not present in the education curricula in neither Serbia nor Kosovo [28], as it is erroneously considered that it is inappropriate and could influence promiscuous behavior. The underlying reason for the lack of sexual education is the general public notion that having sexual education would prompt adolescents and young people into having sexual debut and sexual activity at a younger age, more often and outside of the marital union. In fact, it is extremely difficult to conduct research about reproductive health, STIs and HIV among adolescents and young people, because the initiatives to organize reproductive education courses in schools and health care institutions are not supported by parents and the community. As a result, health care professionals and researchers are insufficiently aware about the level of knowledge on reproductive health among youth and cannot adequately respond to their information needs. As adolescents and young adults are hard to reach in terms of reproductive (sexual) education, it is estimated that they lack the knowledge about condom use and other reproductive health issues. A study conducted in several European countries showed, however, that introduction of sexual education in schools was associated with the reduction of unwanted pregnancies among young people, while the age of sexual debut has not been changed [29].

There is evidence that knowledge does not automatically mean accurate perception of risk [30]. Societal and cultural formatting and individual psychology, may be involved in the perception of HIV risk as well [4]. These factors need to be taken into account, because according to the Behavioral Change Theory, the perceived risk is the focal point to make behavioral changes [4, 5, 31]. To make the actual behavioral change, an accurate perception of risk is required [4, 5]. To be able to achieve this, individuals need appropriate education, but also clear insight into the benefits and barriers to behavioral change [31].

We found some intriguing results regarding attitudes toward PLHIV and their association with self-perceived risk for acquiring HIV. Specifically, students who felt supportive toward PLHIV seem to accept PLHIV and do not fear or stigmatize this population group, which is of paramount importance in the context of Kosovo. As a result, the HIV, as a communicable biological agent, is not being considered as an actual health hazard and they assess their risk as low. This might be the reason as to why the practice of condom use at sexual intercourse is low as well as the uptake of HIV testing among our student. On the other hand, students who fear or are indifferent toward PLHIV were more conscious about HIV risk and, therefore, take precaution and are more willing to take the test. Because of this, they may overestimate their actual risk. Overall, we found that in this study population, attitudes toward PLHIV play much more important role in the assessment of risk, as opposed to actual use of condom or testing uptake.

In the cultural context of Kosovo, STIs including HIV are rarely discussed publicly because of the widespread stigma. Moreover, the fact that there is only one counseling center for STIs in the region, contributes to the finding that the HIV testing rate is low. We observed that taking the test, regardless of the low knowledge was associated with lower perceived risk. This could be interpreted in two ways: increased access to testing facilities might influence the rise in testing rate, which is important for the overall detection of newly infected PLHIV and treatment; however, testing negative may introduce so-called “optimistic bias” [32], where individuals consider themselves at low risk just, because they took the test. Bearing this in mind, the policy makers need to strike a balance between sexual education and access to testing facilities while simultaneously overcoming cultural barriers to topics that should become part of health education. This means that theoretical knowledge needs to be applied in real life situations in which students are aware that they are vulnerable and can be infected. Finally, the discussion about negotiation and actual practice of condom use should be part of sexual education as well.

### Strengths and Limitations

The strength of this study is that it included representative sample of the university students from the Northern Kosovo province. This study is the first of its kind in the Kosovo province and provides unique data specific to the local context. It provides the first insight into the risk perception in this population and we were able to account for several different aspects, such as attitudes toward PLHIV, opinions about high-risk groups, approach to testing and condom use.

A limitation of this study can be the self-assessed risk for HIV, as some studies suggest that self-perceived risk does not accurately represent the actual risk for acquiring HIV [9]. We did not examine the sexual orientation of the study participants, as this might be a sensitive issue with many missing answers. Other data related to testing, condom use etc. were self-reported and as such were open to information bias. Another potential limitation of our study is the fact that we did not examine transaction sex which is known to be linked to the HIV transmission. However, we have not examined this as such questions are seen as sensitive in a conservative culture of Kosovo. This topic would be more suitable to explore within the HIV counseling centers in a qualitative manner, where clients have an established confidential relationship with a counselor. Using simple questionnaires, where many participants provide answers in the same space would not be sufficiently confidential. Finally, this study was designed as cross-sectional, which means that the direction of the association is difficult to establish. Inference about causality is, therefore, limited.

## Conclusion

The majority of students assessed their risk for acquiring HIV as low. Compared to students who assessed their risk as low, lower knowledge about HIV prevention was consistently associated with high and unknown risk for HIV. Students who had low knowledge, but have previously taken the HIV test, assessed their risk for HIV as low. Moreover, being ignorant and indifferent about PLHIV was associated with increased self-perceived HIV risk. These findings highlight the need for continuous specialized HIV-related education to reduce fear and stigma of PLHIV and HIV testing as well as risky behaviors. Further HIV prevention activities are needed in this region, particularly sexual education. This also includes increased opportunities for HIV testing and counseling within the centers for STDs.

### Supplementary Information

Below is the link to the electronic supplementary material.

Supplementary file1 (DOCX 213 KB)

## Data Availability

Data set underlying this study is available on a reasonable request to the corresponding author.

## Abbreviations

HIV: Human immunodeficiency virus
IgnInAt: Ignorance and indifference attitude toward people living with HIV
KnPrev: Knowledge about HIV prevention
KnTrans: Knowledge about HIV transmission
PLHIV: People living with HIV
SD: Standard deviation
SegPrAt: Attitude about segregation and needing protection from people living with HIV
STI: Sexually transmitted infection
SupTrAt: Attitude about support and treatment of people living with HIV
