# Supplementary material for: Risk Perception About HIV Among University Students in One of the Last Hotspots for HIV Transmission in Europe
Source: J Epidemiol Glob Health. 2023 Sep 20;13(4):794–806. doi: 10.1007/s44197-023-00151-y (PMC10686924; doi:10.1007/s44197-023-00151-y)

**RISK PERCEPTION ABOUT HIV AMONG UNIVERSITY STUDENTS IN ONE OF THE LAST HOTSPOTS FOR HIV TRANSMISSION IN EUROPE**

**Supplemental material**

**Supplemental methods.** *Statistical analysis*

**Supplemental Table S1**. Exploratory factor analysis of the knowledge scale about HIV

**Supplemental Table S2**. Exploratory factor analysis of attitude scale towards people living

with HIV

**Supplemental Table S3**. Correlations of students’ self-perceived risk for HIV infection and investigated parameters

**Supplemental Figure S1**. The directed acyclic graph (DAG) model of the association between knowledge about HIV and self-perceived risk for acquiring HIV

***Statistical analysis***

To examine the association of the investigated parameters with self-perceived risk for contracting HIV, we performed the multinomial regression analysis as our outcome variable had three possible responses (low, unknown and high self-perceived risk). The independent variables in the model were only those parameters showing significant correlations with the self-perceived risk for HIV infection. All models were adjusted for two knowledge and three attitude scores. The reference category of the outcome variable was low self-perceived risk and it was compared with unknown and high risk. We observed two models suggesting associations of students' characteristics, knowledge, attitudes, positions and experiences as well as behaviors with the unknown or high self-perceived risk.

We performed the discriminant analysis to determine the parameters that separate two or more groups i.e. outcome categories by constructing discriminatory functions. To assess potential interactions between factors associated with self-perceived risk for contracting HIV infection, we performed the moderation analysis in the SPSS PROCESS. A detailed description of each analysis is provided in the Supplemental material. The obtained significant function adequately predicts the group with highest centroids. Group centroids are means of discriminant function scores according to the group (self-perceived HIV risk) for each calculated function. Parameters that significantly correlate with the function generate the discrimination between groups and are good predictors to which group a case belongs to.

To assess potential interactions between factors associated with self-perceived risk for contracting HIV infection, we performed the moderation analysis in the SPSS PROCESS. Based on the DAG model (Supplemental Figure S1), we hypothesized that higher knowledge levels about HIV transmission and prevention are the most important factors influencing students’ self-perceived risk for HIV, while students’ characteristics could impact this association. The attitude towards PLHIV was considered as a mediator of the association between knowledge about HIV and self-perceived risk for contracting HIV. The variables showing statistically significant association with self-perceived risk for contracting HIV (and were not labelled as confounding factors in the DAG model - red circles), were analyzed as moderators of the association between knowledge about HIV transmission and prevention and self-perceived risk for contracting HIV. The moderation effect was in place if in the product of two variables in the model was significant.

**Supplemental Table S1**. Exploratory factor analysis of the Knowledge scale about HIV

| Knowledge items | Factor loadings | |
| --- | --- | --- |
|  | Factor 1  Knowledge about prevention | Factor 2  Knowledge about transmission |
| Transmission via hand shaking | -0.024 | **0.676** |
| Transmission via sexual intercourse | -0.431 | **0.308** |
| Transmission via sharing personal hygiene products | 0.088 | **0.661** |
| Transmission via sharing cutlery | 0.508 | **0.543** |
| Transmission via equipment for drug injection | -0.515 | **0.422** |
| Transmission via insects | 0.225 | **0.408** |
| Transmission via swimming in pools, sea or rivers | 0.138 | **0.693** |
| Transmission via sharing the room | 0.015 | **0.770** |
| HIV vaccine exists | **0.337** | 0.087 |
| HIV+ person is easy to recognize | **0.397** | -0.003 |
| Just one healthy partner equals no risk | **0.030** | -0.098 |
| HIV+ mother can have a HIV- child | **-0.010** | -0.192 |
| Current therapy enables the usual life expectancy | **0.249** | -0.262 |
| Washing hands reduces the risk of HIV infection | 0.221 | **0.412** |

**Supplemental Table S2**. Exploratory factor analysis of the Attitude scale towards people living with HIV

| Attitude items | Factor loadings | | |
| --- | --- | --- | --- |
|  | **Factor 1**  Segregation and protection | **Factor 2**  Support and treatment | **Factor 3** Ignorance and indifference |
| People with HIV got it because  they deserved it | 0.163 | 0.121 | **0.605** |
| Children should be removed from  HIV + parents | **0.640** | -0.168 | 0.025 |
| People with HIV have same rights as others | 0.102 | **0.447** | -0.042 |
| Persons who got HIV by transfusion deserve better treatment | 0.024 | -0.184 | **0.626** |
| HIV have only injecting drug users, homosexual persons of both genders and sex workers | 0.161 | -0.057 | **0.659** |
| Employers have right to let off HIV + persons | **0.622** | -0.150 | -0.011 |
| All medications for HIV should be free | 0.110 | **0.390** | -0.177 |
| Testing for HIV should be enforced by the law | **0.078** | -0.514 | -0.126 |
| HIV + and - children should go to  different schools | **0.688** | -0.007 | -0.031 |
| I would not worry for my health if  a colleague had HIV | -0.491 | **-0.092** | -0.124 |
| Employers should know if their  colleague has HIV | **0.137** | -0.631 | 0.056 |
| HIV + people should be isolated | **0.669** | 0.076 | 0.038 |
| I would take care of a family member  with HIV | 0.050 | **0.407** | 0.187 |
| I would not want that everyone knows  the HIV status of my family member | -0.269 | -0.129 | **-0.038** |
| I do not want to contact or communicate  with HIV + people | **0.521** | -0.014 | 0.050 |
| Paying for sex is acceptable | -0.034 | **0.420** | 0.027 |
| HIV/AIDS are not a health problem  in our country | -0.065 | 0.119 | **0.299** |

**Supplemental Table S3**. Correlations of students’ self-perceived risk for HIV infection and investigated parameters

| Parameters | | Self-perceived risk  for HIV infection | Parameters | Self-perceived risk  for HIV infection |
| --- | --- | --- | --- | --- |
| Students’ gender | rho | **0.168** | Opinion on  drug addicts | -0.009 |
|  | p | **0.001** |  | 0.776 |
| Students’ age | rho | -0.014 | Opinion on homosexual persons | **-0.083** |
|  | p | 0.657 |  | **0.008** |
| School groups | rho | -0.009 | Opinion on  sex workers | 0.001 |
|  | p | 0.768 |  | 0.986 |
| Study year | rho | 0.022 | Clubbing frequency | 0.046 |
|  | p | 0.483 |  | 0.145 |
| Extra activities  (employment) | rho | 0.023 | Alcohol drinking | 0.060 |
|  | p | 0.456 |  | 0.056 |
| Relationship status | rho | 0.043 | Opioids use | -0.029 |
|  | p | 0.174 |  | 0.350 |
| Residence  during schooling | rho | 0.052 | Knowledge of HIV transmission | **0.114** |
|  | p | 0.096 |  | **0.000** |
| Previous HIV testing  (yes/no) | rho | **0.063** | Knowledge of HIV prevention | **0.102** |
|  | p | **0.046** |  | **0.001** |
| HIV testing experience  and reasons | rho | **-0.065** | Media sources  for information | -0.014 |
|  | p | **0.037** |  | 0.660 |
| Approach to testing (positive/negative) | rho | **-0.064** | Internet sources  for information | -0.040 |
|  | p | **0.042** |  | 0.197 |
| Previous contact  with PLHIV | rho | 0.003 | Medical source of information | 0.008 |
|  | p | 0.936 |  | 0.802 |
| Actions after learning  ones’ HIV status | rho | **0.109** | Information from  university professors | -0.050 |
|  | p | **0.001** |  | 0.111 |
| Condom use in most  recent intercourse | rho | **0.079** | Information  from friends | 0.021 |
|  | p | **0.012** |  | 0.510 |
| Condom use with  casual partner | rho | **0.101** | Special HIV education | -0.021 |
|  | p | **0.001** |  | 0.513 |
| Support and /or treatment attitude | rho | **-0.110** | Sex partners number  in last year | **0.133** |
|  | p | **0.001** |  | **0.001** |
| Segregation and/or protection attitude | rho | 0.060 | Sexually transmitted  diseases in last year | 0.014 |
|  | p | 0.056 |  | 0.659 |
| Ignorant and/or indifferent attitude | rho | 0.029 | / | / |
|  | p | 0.348 |  |  |

**Supplemental Figure S1**. The directed acyclic graph (DAG) model of the association between knowledge about HIV and self-perceived risk for acquiring HIV


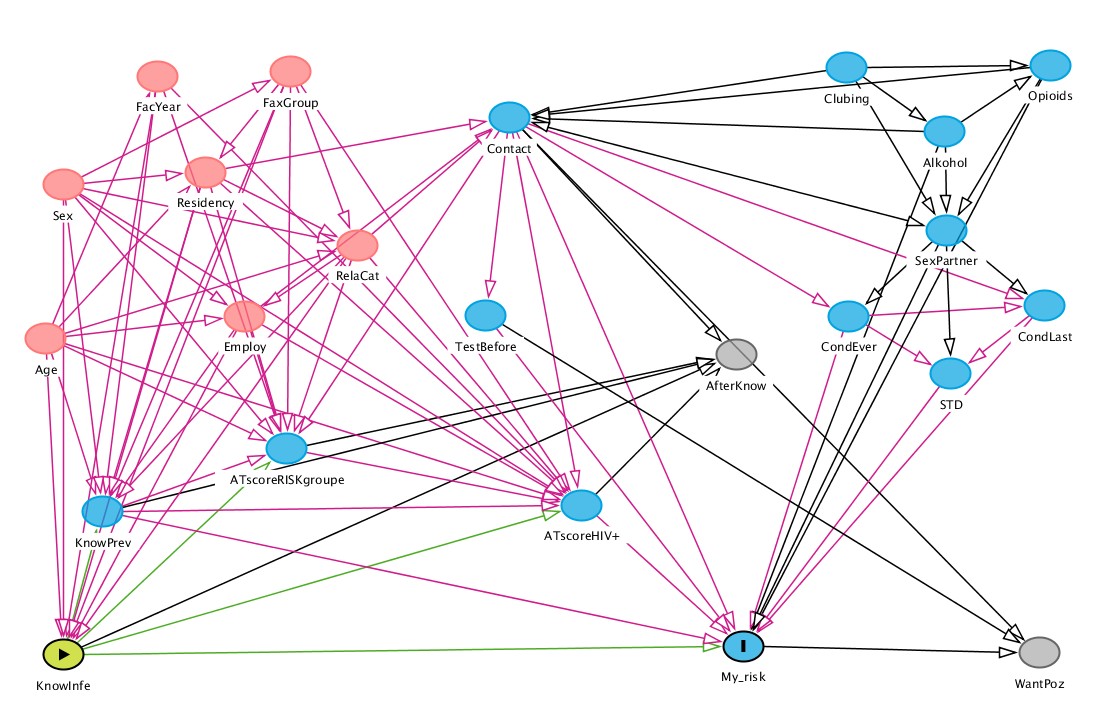

Supplement: Supplementary file 1 — Supplementary file1 (DOCX 213 KB) [file 44197_2023_151_MOESM1_ESM.docx]
